# A qualitative study of experiences among young adults who increased their cannabis use during the COVID-19 pandemic

**DOI:** 10.1186/s12889-024-19886-9

**Published:** 2024-09-06

**Authors:** Laura L. Struik, Alexia Armasu, Genevieve Fortin, Teodora Riglea, Jodi Kalubi, Olivier Ferlatte, Mounia Naja, Jennifer O’Loughlin, Marie-Pierre Sylvestre

**Affiliations:** 1 Faculty of Health and Social Development, School of Nursing, University of British Columbia, Okanagan Campus1628 Dickson Ave., Landmark 4 – 609, Kelowna, BC V1Y 9X1 Canada; 2 Centre de recherche du Centre hospitalier de l’Université de Montréal (CrCHUM), Montréal, QC Canada; 3 Department of Social and Preventive Medicine, School of Public Health, Université de Montréal, Montréal, QC Canada; 4 Centre de recherche en santé publique (CReSP), Montréal, Québec Canada

## Abstract

**Background:**

Young adults face unique vulnerabilities during major life disruptions like the COVID-19 pandemic. The pandemic contributed to increases in mental health challenges and substance use among young adults. This study explores the experiences of young adults who increased their cannabis use during the pandemic.

**Methods:**

Participants were recruited from the Nicotine Dependence in Teens (NDIT) study, and qualitative data were collected through semi-structured interviews conducted via Zoom. A total of 25 participants (ages 33–34) reporting increased cannabis use during the pandemic were included. Thematic analysis and gender-based analysis was employed to extract key themes.

**Results:**

Five themes emerged: (1) No disruption in cannabis use; (2) Cannabis use to manage declines in mental health; (3) Cannabis use to break up pandemic boredom; (4) Cannabis use as an expression of freedom; (5) Cannabis use as “another way to chill out.”

**Conclusions:**

This research provides valuable perspectives on how major life disruptions, like the COVID-19 pandemic, influence cannabis use among young adults. The findings offer guidance for public health initiatives and highlight avenues for further investigation.

**Supplementary Information:**

The online version contains supplementary material available at 10.1186/s12889-024-19886-9.

## Introduction

The COVID-19 pandemic represented a major life disruption with unexpected changes to family dynamics (e.g., everyone at home), routines (e.g., school and extracurricular facilities closed), socialization (e.g., closure of restaurants and cancellation of social events), and employment (e.g., working at home and job loss) [1, 2]. Because young adults are in a transitional life stage and often in the process of establishing themselves in these areas, they were particularly vulnerable to these disruptions, [3] and were among those most vulnerable to increasing substance use and mental health challenges in response to pandemic-induced distress [4, 5]. A global cross-sectional survey of 1,653 participants ages 18–82 found that younger adults (age 18–34 years) were more vulnerable to pandemic-related stress, anxiety, and depression than older age groups [6].

Some evidence suggests that pandemic-related distress could have prompted increases in cannabis use to alleviate these negative effects [1, 7, 8]. For example, the Canadian Cannabis Survey in 2021 reported that 29% of Canadian cannabis users increased their cannabis use during the pandemic, which users attributed to boredom, stress, anxiety, lack of a regular schedule, and loneliness [9]. This appears to be especially true among young adults. According to a recent longitudinal study of changes in substance use before and during the pandemic, cannabis was the only substance that showed an increase in initiation and an increase in weekly/daily use among young adults age 30. [10] The experiences of young adults who increased their cannabis use during the pandemic to contextualize these numbers, however, remain unexplored.

There is emerging evidence that gender influences cannabis use. According to a systematic review, males are more likely to conform to “male typicality”, and are at higher risk of increased use during young adulthood than females [11]. In addition, gendered norms play different roles, with some protective of substance use (e.g., women are nurturing) and others conducive to substance use (e.g., women are relational) [11], lending to the conclusion that more more research is needed to understand the role of gender in cannabis use specifically. Some research has explored the role of gender in cannabis use during the pandemic. Compared to men, women reported greater increases in cannabis use to manage mental health challenges during the pandemic [12–15]. This may be due to women reporting higher rates of mental health issues during the pandemic compared to men [16]. Understanding the experiences of women and men during the pandemic is needed to unpack the relationship between gender and increased cannabis use during the pandemic.

Because cannabis was legalized in Canada in 2018, the pandemic paralleled rapid expansion of the cannabis market in cannabinoid composition (i.e., THC and CBD), potency of cannabis products, and delivery formats (e.g., inhalation, oral, topical) (17–18), which prompted an increase in cannabis use among young adults in particular [18–20]. Because most provinces and jurisdictions in Canada deemed cannabis retailers an essential service during the pandemic, [9] retailers were able to adapt by offering curbside pickup or online sales to reduce in-person interactions [21]. Understanding how such changes impacted cannabis use during the pandemic needs exploration.

Thus, there are numerous gaps in our understanding of cannabis use experiences among young adults during the pandemic. In this study, we addressed these gaps in an exploratory qualitative study of young adults who increased their cannabis use during the pandemic. Our objectives were to: (i) describe the cannabis use experiences of young adults during the pandemic; and (ii) investigate gender differences in their cannabis use experiences. Understanding these experiences is critical to informing the development of effective interventions to reduce the harms of cannabis use, particularly during major life disruptions.

## Methods

### Context – NDIT study

Participants were recruited from the Nicotine Dependence in Teens (NDIT) study, an ongoing investigation of 1294 participants recruited in 1999–2000 at ages 12–13 (grade 7 students). NDIT initially aimed to investigate the natural course and determinants of cigarette smoking and nicotine dependence in adolescence, [22] but quantitative data collection expanded to encompass use of other substances, lifestyle behaviours, genetics, anthropometrics measures, psychosocial factors, and mental health. Between 1999 and 2005 when participants (ages 12–17) were in high school, they completed self-report questionnaires every 3 months in a total of 20 data collection cycles. After high school, five additional data collection cycles have taken place to date (i.e., cycle 21 in 2007-08 at mean age 20.4 (SD = 0.8); cycle 22 in 2010-12 at mean age 24.0 (0.7); cycle 23 in 2017–2020 at mean age 30.6 (1.0); cycle 24 in 2020 -21 during the COVID-19 pandemic at mean age 33.6 (0.8) ; and cycle 25 at mean age 35.2 (0.61).

NDIT was approved by ethics committees at the Montreal Department of Public Health, McGill University and Centre hospitalier de l’Université de Montréal (2007–2384, 2017–6895, ND06.087). Parents/guardians provided written informed consent at baseline and participants, all of whom had attained legal age, provided consent post-high school.

### Data collection

Participants who reported any increase in cannabis use in the past year during cycle 24 were eligible to participate in this qualitative study. From July to September 2021, a purposive sample of eligible English- and French-speaking males and females were invited to participate in 60-minute semi-structured interviews conducted via Zoom by AA and GF. During these interviews, participants were asked about how the pandemic influenced their mental health and health behaviors, including cannabis use. Herein, we present data on cannabis use during the pandemic among these young adults. These data are based on seven interview questions, including type, frequency, and context of use during the pandemic, reasons for use during the pandemic, and perceived positive and negative impacts of the pandemic on cannabis use.

### Data analysis

All interviews were audio-recorded and transcribed by a third party. All transcripts were reviewed by research assistants for accuracy and then anonymized. We disaggregated the data by gender and analyzed the data using Braun and Clark’s thematic analysis approach [23, 24], which comprises of six phases (familiarisation, initial code generation, theme generation, theme review, theme confirmation, and reporting). We positioned our analysis within a constructivist epistemology and interpretive/subjectivist ontology, meaning that the themes were developed through communication with the participants and through interpretations by the researchers. For phase one (familiarisation with the data), three researchers (GF, LS, and AA) read the transcripts and collaboratively iterated on what they were seeing in coding meetings. For phase two (initial code generation), we employed Nvivo qualitative data analysis software to generate descriptive codes for responses to the interview questions. For example, if a participant mentioned using cannabis to treat heightened anxiety during the pandemic, we created a broad code of “mental health” and then a sub-code of “anxiety”. One researcher (LS) then coded the transcripts for women, and another (AA) coded the transcripts for men (so that gender-based influences on cannabis experiences could be identified). During phase three (theme generation), after all transcripts were coded, we engaged in collaborative theme development meetings, wherein we discussed what meaning was shared across the entire sample and what meaning was unique to women or men as a collective, or even to individual participants. We did this iteratively until we established meaning saturation and no new themes emerged when reviewing the coded data. During phase four (theme review), we collaboratively iterated on the themes to ensure that nuances were appropriately captured. For phase five (naming themes), we discussed and generated a thematic framework to capture the themes and their relationships to each other.

### Rigor

The credibility of the study is established through content and methodological expertise among the study team members, and through the development of a codebook. The dependability of the study is ensured through a data analysis audit trail that we kept track of via research team reviews and summaries of transcripts, use of collaborative coding, and use of Nvivo data analysis software. The confirmability of the study findings is ensured through the frequent team meetings that were held to discuss and confirm consensus of the meaning of the findings throughout data analysis. The transferability of the study findings is enhanced through the robust sample size included in this study, with equal representation of males and females.

## Results

### Sample

A total of 25 participants were included in this study (Table 1). Participants were all age 33 or 34, and just over half were female. All female participants identified as women and all male participants identified as men. All participants reported using cannabis daily or weekly in the past year. Most had more than high-school education in both female and male samples (91.7% and 84.6% respectively), and a household income above $50,000, which was slightly higher among males at 74.9% compared to 53.9%. Except for one, all participants were born in Canada. More female participants reported living alone (46.2%), being unemployed (38.5%), and having a history of a mental health diagnosis (53.8%). More male participants reported past year substance use of any kind compared to females. More females scored higher on depressive and anxiety symptoms than males.

**Table 1:** Characteristics of participants in the qualitative interviews (*n* = 25) according to sex, nicotine dependence in teens study, 2020–2021

	Men (*n* = 12)	Women (*n* = 13)
Age, years, mean (SD)	33.8 (0.5)	33.5 (0.6)
More than high school education, %	91.7	84.6
Annual household income > $50,000, %	74.9	53.9
Born in Canada, %	91.7	100.0
Lives alone, %	25.0	46.2
Unemployed, %	16.7	38.5
Any use in the past year…% Cannabis^a^ Alcohol Binge drinking Combustible cigarettes E-cigarettes	100.0 100.0 83.3 33.3 25.0	100.0 92.3 76.9 30.8 15.4
History of mental health diagnosis, %^b^	25.0	53.8
Depressive symptoms (MDI)^c^, mean (SD)	12.6 (10.0)	18.5 (9.8)
Anxiety symptoms (GAD-7)^d^, mean (SD)	4.5 (4.5)	9.8 (6.6)

a.Participants were eligible for inclusion in the study if they reported any cannabis use in the past year.

b.History of mental health diagnosis was measured as diagnosis at any of cycles 21, 22, or 23. This variable was not measured in cycle 24.

c.Major Depression Inventory (MDI). [25]

d.Brief Measure of Generalized Anxiety Disorder (GAD-7). [26]

### Qualitative themes

Our findings revealed five themes that capture the influence of the pandemic on young adult cannabis use including: (1) no disruption in cannabis use; (2) cannabis use to manage declines in mental health; (3) cannabis use to break up pandemic boredom; (4) cannabis use as an expression of freedom; and (5) cannabis use as “another way to chill out”. Gender-specific findings in relation to these themes were identified.

### No disruption in cannabis use

Men reported cannabis use as a more regular day-to-day activity than females. Thus, men were particularly vocal about experiencing little or no disruption in their use due to pandemic restrictions and lockdowns. Men reported that access to cannabis during the pandemic was not only unhindered, but became even easier. In addition to cannabis stores being considered an essential service in Quebec and allowed to remain open, these outlets quickly adapted to alternative and convenient modes of delivery, such as home delivery. Men reported enhanced accessibility as a major facilitating factor for use during the pandemic:

I mean already there was the accessibility. That’s one issue, that you can just go to the store and pick it up at any time. And it’s essential so it doesn’t close, while everything else does. (male, 1070001)

‘Cause if you ordered before 1:00 PM on their website, you would get it that evening. So I would literally like order it during the day and then at night, I’d order Uber Eats and my food and my weed would just get delivered to my door [laughs]. It was like “The future is here and it’s great”! [laughs] So yeah, that definitely contributed to like the more, easier access to it, like I didn’t even have to go to the store and have to deal with waiting in line, waiting in a huge line. Uh… and then going in the store, wearing a mask, all that stuff. (male, 115003)

#### Cannabis to manage declines in mental health

Participants frequently reported an increase in cannabis use during the pandemic because they struggled with heightened anxiety and stress in response to the impacts of the pandemic including job loss, working from home, having no childcare, and the overall stress of lockdowns:

Maybe [I used] a little bit more in terms of the anxiety that was coming up. So maybe I had a lot more bad nights during the pandemic than I would have had if there had been no pandemic. (female, 2580010)

First time [I used cannabis] was just like curiosity and just kind of for fun. And the second time was COVID. That is actually the reason. (male, 80002)

Cannabis use to cope during the pandemic was particularly prevalent among women who were mothers. Women spoke to decreases in their mental health due to job-related stress combined with increased childcare responsibilities. They often described feeling out of control, and using cannabis was a way to cope with that feeling:

The biggest stressors for me were like the kids being home the whole time, or like for that stretch from March till the end of August of last year. And my work situation, just really feeling completely out of control. (female, 350001)

Many women, however, emphasized that they were using cannabis as an alternative to managing their mental health symptoms rather than to get high. Women with children made sure to emphasize that they did not use it in front of their children:

Well, a lot of times it’s when I have my kids my stress level is really, really high. So, around like 6 o’clock, I can’t take it anymore, so I go and smoke like three four puffs in the garage. (female, 1860010)

In addition, several women reported that endorsement by their doctor was important to their decision-making and comfort in using cannabis to treat their mental health symptoms:

[My Dr.] was like: “That’s good… if you can find something … to not be on Ativan every day instead.” (female, 1660001).

#### Cannabis as a way to break up pandemic boredom

Participants described feelings of boredom during the pandemic, which was largely blamed on their inability to engage in their usual day-to-day activities (e.g., going to work, gyms, or restaurants; socializing; participating in extracurricular activities). As a result, they described cannabis use as a tool that enabled them to face boredom and provided a means to entertain oneself during lockdowns and stay-at-home orders:

I think, it’s really just boredom, during the pandemic, not [laughs]-not having any action, like, it’s-it’s just that, basically. It wasn’t like, “Oh, my God, I’m sad right now, I want to like forget about it.” No, it was just like, “This is boring! Let’s try some things to like get a little buzz, a little effect.” (female, 720006).

In this vein, participants described using cannabis as something that became popular amongst a variety of social circles during the pandemic. They explained that all the new compositions (e.g., THC/CBD) and formats (e.g., edibles) of cannabis that were made widely available following the legalization of cannabis in Canada, prompted them to try new modes of consumption and new products because there was a sense among participants that cannabis was more acceptable and that everyone was trying it and discussing it:

During the pandemic, there was this new trend with candy. And I saw some very serious people - well, not very serious, but you know, very functional people, I mean, who work, who do, uh… who were talking to me about it, like, “Oh yeah, it’s really cool. It helps you relax.” So I tried it. I ordered a bottle-uh, a bottle-uh yeah, that’s right, a little bottle of candy. And yeah [laughs]! I thought it was like chill to try like at night. I was like, “Ah, this is cool! You know it’s relaxing.” Then I was taking something too that was- there was more- what do you call it? CBD… (female, 720006).

Cannabis use was also used to maintain social routines to combat pandemic boredom. One participant described smoking cannabis during video chats as a way to keep up his social connections:

So it was still kind of a social thing ‘cause I would be smoking in my room while my friends were on video calls, also smoking weed. So it’s like almost like we were doing the same thing that we were doing before, so it was– there was a little bit of social element. But again, it was only like once a week, but I was still smoking every day. (male, 1150003)

#### Cannabis use as an expression of freedom

Some participants, especially women, described cannabis use as an expression of freedom from the lockdowns. Nearly all participants reported first trying cannabis during adolescence. Therefore, using cannabis during the pandemic was paralleled with memories of when they were young and free. They described feeling like teenagers again, and wanting to be a bit wild when they felt so inhibited by the lockdowns. Some described using during lockdowns with others in their bubble, while others described using when there were breaks in the lockdowns at social gatherings as a way to celebrate:

We had gone out one night, and I felt like a teenager again. One of my friends had some edibles in the freezer, so me and my friends were like, “let’s try it”. (female, 710001)

Uh, well I think, uh, there’s a kind of feeling like, “We got our freedom back”, you know. I must have been feeling a bit wild there you know like “anything goes” [laughs]! (female, 2090010)

#### “Another way to chill out”

Many participants reported using cannabis as a way of winding down from their hectic days, often referred to as an alternative to winding down with a glass of wine:

Oh I guess– I guess when I was using it, I was just kind of thinking…this is another way I can chill out. Like, can I do this or can I replace this with a glass of red wine, kind of thing? (female, 450003)

Some participants talked about how their alcohol consumption increased during the pandemic. They concluded that cannabis was a better alternative since they did not suffer from the side effects of excessive consumption like they did with alcohol (e.g., hangover):

Because initially…in the pandemic, it was more like let’s have drinks every night and that I feel has more of an effect on my state the following day, with a hangover, or it triggers a migraine. So I avoid that with cannabis. Which is I guess the lesser of two evils. (female, 350001)

While women reported using cannabis to wind down usually after tasks were completed and kids asleep, men often reported using cannabis earlier in the evening and as something to help them connect and feel more present with their family:

Like I don’t smoke a lot, you know I’d be like two or three puffs – and it really helps me to calm down and be present with my kids. (male, 420002)

Further, some women reported smoking cannabis as a way to connect with their husband or partner while they were winding down from the day. In fact, several women reported that they only started using it because their partner was using it:

Like I think for… in terms of smoking it, it just became… it was occasionally something to do cause my husband’s smoking it and… it was a nice way to like wind down in the evening kind of thing…it was enjoyable to do when it was… warm at night and we could sit outside and we would talk and we could you know smoke a little bit and chat and stuff like that. (female, 1660001)

## Discussion

### Main findings

This study examined contextual factors that influenced cannabis use among young adults who reported an increase in cannabis use during a major life disruption, the COVID-19 pandemic. Themes in this study revealed that this disruption was associated with uninterrupted access to cannabis, a way to manage mental health, something that helped them manage boredom, something that reminded them of when they were young and free, and as another way to wind down. The findings also revealed some gender-based differences in use, whereby women expressed childcare and job-related strain as a key factor in increasing cannabis use. In addition, women were adamant about not exposing their children to cannabis, not using it to get high, and receiving their doctor’s approval to use cannabis to manage mental health symptoms. Finally, some women reported smoking cannabis (versus using oils or edibles) as a way to connect with their male partner.

Similar to other studies [8, 27, 28] young adults in this study reported increasing use to help them cope throughout the pandemic. For example, Clendennen et al. [27] found that increases in stress and symptoms of anxiety and depression predicted increases in cannabis use among young adults during the pandemic. Noteworthy in our study was the strain that mothers experienced. This may be due, in part, to the fact that childcare fell primarily on women during the pandemic [29]. According to a survey in the United States, compared to fathers, mothers reported that they were more likely to face professional hurdles, reduce their work hours, face difficulties in getting work done, and shoulder most of the caregiving burden [30]. The experiences of mothers in this study suggest a disproportionate impact of the pandemic on them regarding childcare, which could be attributed to normative gender-based roles (e.g., women are caregivers) that appeared to naturally fall into place when supports for women and working mothers (e.g., daycare) were no longer available. Our findings thus confirm that the lack of much needed targeted relief for mothers (e.g., social support, employment support) is an area for development.

Supportive of other studies, [31] young adults reported that cannabis use helped ease boredom during the pandemic. With limited opportunities for socialization, and the inability to engage in recreational activities that were part of their typical routine (e.g., going to the gym), young adults in this study used cannabis to break free and remedy their boredom. This method of coping with boredom raises concerns about the long-term impacts of the pandemic on substance use in general, including cannabis use behavior. It also raises questions about how young adults adapt to disruptions over time and if the increase in use in response to boredom was temporary. Regardless, these findings underscore the importance of recreational and social outlets for the young adult population.

The pandemic followed legalization of cannabis in Canada in 2018 closely [21], with new cannabis formats and potency options more readily available during the pandemic. This may have contributed to participants likening their cannabis use during the pandemic to their first use as teenagers, whereby new product options offered renewed novelty and excitement around cannabis. Similar to other studies [32, 33], the effects of the pandemic on nostalgia for the “good old days” was expressed through engaging in activities that reminded participants of times when they were young and free (e.g., listening to music). Although there is evidence that nostalgia is protective of mental health and that it promoted happiness during the pandemic [34, 35], the effects of cannabis use as a nostalgic activity needs more investigation.

The influence of gender on differential use and perceptions of cannabis was noteworthy. In particular, women appeared to be attuned to stigmatization associated with their use, emphasizing that they would not use around their children, were using it primarily to ease mental health symptoms rather than to get high, were more likely to use formats that did not produce smoke (e.g., oils and edibles), and reported that their doctor’s approval was important to them. Despite that legalization of cannabis has somewhat normalized cannabis use, social norms that associate cannabis use with masculinity problematizes cannabis use among women [36]. In an analysis of cannabis stigmas, Reid [37] cautioned against claims of normalization of cannabis use since qualitative accounts reveal increasing evidence that social stigmas are intersecting with particular groups who typically experience inequality, including women. Our qualitative findings underscore women’s accounts that reflect a desire to resist stigmatization of their use.

It was also interesting how cannabis use among men influenced uptake among their female partners. Women reported joining their partner in using cannabis as a way to connect and spend time together. Researchers examining heterosexual couples found that joint cannabis use appears to strengthen relationship functioning in the short term [38]. However, there is a gap in the literature regarding the impact of cannabis use among couples in the long term, as well as among couples whereby one partner started using cannabis because of the other partner.

### Implications and future research

This study has implications for public health and future research. In relation to public health, the findings pinpoint actions that can be considered to support young adults through major life disruptions, like the COVID-19 pandemic. For example, promoting social and extracurricular outlets for young adults (e.g., discounts on camping or park passes), supporting mothers with childcare costs and relief (e.g., funding, space), creating supports in the context of job insecurity (e.g., lobby for government grants that could be activated during a global pandemic), and ensuring that doctors are educated on the effects of cannabis use should be a priority. Additionally, promoting healthy outlets to express nostalgia (e.g., through music or activities) may also be a productive way to promote resilience and happiness through challenging disruptions. In relation to future research, understanding the temporality of increased cannabis use during the pandemic, the potential benefits and pitfalls of using cannabis as a nostalgic activity, and the influence of partners on cannabis use are all areas which will benefit from future research.

### Strengths and limitations

A strength of this study is that it is one of the first to explore how the pandemic influenced cannabis use among young adults through the voices of young adults. Another strength is the sample size and representation of women and men. As a result, a novel contribution of this study is an exploration of gender differences in the experience of cannabis use during the pandemic. A strength is also that the sample was drawn from a large pool of young adults in Quebec, Canada. Finally, the findings provide helpful directions for future research, and directions for public health advocates to consider when supporting young adults during major life disruptions, such as the pandemic.

Limitations include that this study involved a Quebec sample and Quebec had different rules around COVID-19 than other provinces, possibly limiting transferability of the findings. Another limitation is that the interviews were conducted in the summer of 2021, such that the full impact of the pandemic may not have been captured. However, the interviews were conducted after the lockdown, which represented a critical timeline during the pandemic. In addition, pandemic regulations during the summer, and access to the outdoors due to the weather, may have influenced participants’ cannabis use. Usage patterns and perceptions on cannabis use may have shifted after this time point (e.g., some users may have decided to quit or commit to a decrease in use). Finally, we did not measure the level of increase in cannabis use in this study sample.

## Conclusion

This study examined how a major life disruption, the COVID-19 pandemic, influenced cannabis use among young adults. Our findings reveal the contextualized experiences of young women and men in navigating such a disruption and how it impacted their cannabis use. Young adults found that they did not experience any disruption in their ability to access cannabis, and they described increasing their use due to mental health struggles, feeling bored, as an expression of freedom during restrictions, and as a way to relax. These findings have important public health implications and identify key areas for future research.

## Electronic supplementary material

Below is the link to the electronic supplementary material.

Supplementary Material 1

## Data Availability

“Data can be accessed by completing a data access form, which is available on the Nicotine Dependence in Teens website (www.nditstudy.ca), and will then need to be submitted to the NDIT principal investigator, Dr. Jennifer O’Loughlin (jennifer.oloughlin@umontreal.ca). “.
